# Editorial: Fungal virulence

**DOI:** 10.3389/ffunb.2024.1530202

**Published:** 2025-01-03

**Authors:** Jaime David Acosta-España, Antonio José de Jesus Evangelista, Jonathas Sales de Oliveira, Rosana Serpa

**Affiliations:** ^1^ Health Sciences Faculty, Universidad Internacional SEK (UISEK), Quito, Ecuador; ^2^ School of Medicine, Pontificia Universidad Católica del Ecuador, Quito, Ecuador; ^3^ Institute of Microbiology, Friedrich Schiller University Jena, Jena, Germany; ^4^ Research Group of Emerging and Neglected Diseases, Ecoepidemiology and Biodiversity, Health Sciences Faculty, Universidad Internacional SEK (UISEK), Quito, Ecuador; ^5^ Centro de Investigación para la Salud en América Latina (CISeAL), Pontificia Universidad Católica del Ecuador, Quito, Ecuador; ^6^ Clinical Mycology in the Biomedicine Course, Centro Universitário Christus (UNICHRISTUS), Fortaleza, Ceará, Brazil; ^7^ Microbiology, Immunology and Parasitology, School of Medicine, Institute of Medical Education (IDOMED), Canindé, Ceará, Brazil; ^8^ Researcher at the Virulence and Microbial Pathogenicity Research Group, Federal University of Ceará (UFC), Fortaleza, Ceará, Brazil; ^9^ Federal Institute of Education, Science, and Technology of Ceará, Fortaleza, Ceará, Brazil; ^10^ Department of Microbiology and General Biology, Federal Institute of Education, Science and Technology of Rio Grande do Sul, Viamão, Rio Grande do Sul, Brazil; ^11^ Coordination of the Higher Technological Course in Environmental Management, Federal Institute of Education, Science and Technology of Rio Grande do Sul, Viamão, Rio Grande do Sul, Brazil

**Keywords:** fungal infections, virulence factors, thermotolerance, climate change, pathogenic fungi

## Introduction

Fungi are critical components of terrestrial ecosystems, participating in nutrient cycling, soil formation, and plant growth promotion (Abu Bakar et al., 2020; Nnadi and Carter, 2021; Seidel et al., 2024). However, the rising prevalence of fungal pathogens poses significant risks to human and veterinary health and agriculture (Ni et al., 2024; Seidel et al., 2024). Recent data indicate a disturbing increase in fungal infections, particularly among immunocompromised individuals and those with pre-existing health conditions. For instance, the World Health Organization reports that invasive fungal diseases affect millions globally, with mortality rates exceeding 50% in some cases (Casadevall, 2019; World Health Organization, 2022).

The concept of virulence factors is pivotal in understanding the pathogenicity of fungi (Hogan et al., 1996). Thermotolerance is the ability to survive and thrive at elevated temperatures and is becoming increasingly significant due to climate change. As global temperatures rise, previously non-pathogenic fungi may adapt to infect humans and animals, raising serious public health concerns (Abu Bakar et al., 2020; Casadevall, 2020; Ni et al., 2024; Seidel et al., 2024). Therefore, understanding the mechanisms that confer virulence, particularly thermotolerance, is essential for developing strategies to combat fungal infections.

This Research Topic reviews recent findings on fungal pathogenesis from the “*Fungal Virulence*” Research Topic of Frontiers in Fungal Biology. It focuses on the regulatory mechanisms of virulence in fungal pathogens. Examining these mechanisms provides insights into fungi’s adaptive strategies in response to environmental changes, which could inform clinical practices and public health initiatives.

## 11 Regulatory role of Mssin *Candida glabrata* virulence

Wang et al. have shown the critical role of the transcription factor *Mss11* in regulating virulence in *Candida glabrata*. Unlike other pathogenic fungi, *C. glabrata* does not exhibit characteristics like pseudohyphal growth or significant protease secretion. Instead, it relies heavily on its adhesive capabilities, primarily facilitated by epithelial adhesins (Epas) and biofilm formation. The study demonstrated that *Mss11* is essential for expressing adhesin genes *EPA1* and *EPA6*, which are crucial for adherence to host tissues.

Experiments with *Mss11* deletion strains showed significantly impaired cell surface hydrophobicity, a key factor for adherence to epithelial cells and abiotic surfaces. *In vivo* assays using a *Galleria mellonella* infection model revealed that *Mss11* deletion reduced virulence, underscoring its importance.

Molecular analyses indicated that *Mss11* binds to the promoter regions of adhesin genes and those involved in subtelomeric silencing (*SIR4*, *RIF1*, *RAP1*). This dual regulatory function highlights the complexity of fungal virulence, suggesting that targeting *Mss11* could be a promising therapeutic strategy to disrupt *C. glabrata* ability to adhere and form biofilms.

## Cell wall composition in *Cryptococcus neoformans* varies with media, affecting host response and inducing immunity

Upadhya et al. focused on the influence of growth medium on *Cryptococcus neoformans* virulence. The researchers assessed how different culture conditions affect cell wall composition and immunogenic properties. By comparing cultures in yeast extract-peptone-dextrose (YPD), yeast nitrogen base unbuffered (YNB-U), and YNB buffered to pH 7 (YNB, pH 7), they found that growth conditions significantly influenced chitosan levels within the cell wall.

Chitosan is vital for maintaining cell wall integrity and virulence. Notably, cells grown in YNB-U exhibited lower chitosan levels, resulting in structural disruptions and increased immune recognition. These YNB-U-grown cells were avirulent in murine models, contrasting with those cultivated in YPD or YNB, pH 7, which retained virulence.

Additionally, the immune response to heat-killed YNB-U-grown cells was characterized by severe inflammation and early mortality in infected mice due to a hyper-inflammatory lung response marked by elevated levels of pro-inflammatory cytokines. Conversely, cells grown in YNB, pH 7, triggered a protective immune response when used as a vaccine, emphasizing the importance of growth conditions in modulating immune responses and informing vaccine development.

## Environmental influences on *Macrophomina phaseolina* pathogenicity

Pamala et al. looked at *Macrophomina phaseolina*, a pathogen affecting groundnut crops, highlighting the relationship between environmental conditions and virulence. This study examined pathogenic variability across Southern India, correlating climatic factors with disease incidence. Increased temperatures and reduced rainfall were associated with a higher incidence of dry root rot caused by *M. phaseolina*, raising concerns for agricultural production and food security.

Surveys across various groundnut-producing regions revealed disease incidence rates ranging from 8.06% to 20.61%. The research demonstrated that sandy soils and rain-fed cultivation systems exhibited higher infection rates than irrigated fields with clay-rich soils. Pathogenic variability among isolates was significant, with isolates categorized into four levels of pathogenicity. Morphological and molecular characterization revealed high genetic diversity, which enhances the pathogen’s adaptability and virulence.

## GPI-anchored proteins in *Paracoccidioides brasiliensis*: a novel diagnostic avenue

Gonçales et al. looked at the immunogenic potential of glycosylphosphatidylinositol-anchored proteins (GPI-APs) in *Paracoccidioides brasiliensis*, providing insights into fungal pathogenesis and diagnostic development. The study identified 46 putative GPI-APs within the *P. brasiliensis* genome, with varying expression across different morphological forms.

Four GPI-APs were selected for their surface localization and differential expression, and their immunogenicity was tested with sera from patients with paracoccidioidomycosis. All four proteins elicited immune responses, with some showing significantly higher reactivity compared to sera from healthy controls. These findings suggest that GPI-APs may serve as novel diagnostic markers, warranting further validation in larger patient cohorts.

Understanding the role of GPI-APs enriches our knowledge of host-pathogen interactions, paving the way for improved diagnostic methods and therapeutic strategies against paracoccidioidomycosis.

## Discussion

The studies presented herein underscore the adaptability and complexity of fungal pathogens in response to environmental changes, with significant implications for public health and agriculture. The regulatory mechanisms governing virulence factors, such as thermotolerance, adhesion, and biofilm formation, are crucial for understanding fungal infections (Hogan et al., 1996).

As climate change continues influencing fungal biology, emerging pathogens pose increased human and animal health risks (Casadevall, 2020; Seidel et al., 2024). Therefore, interdisciplinary approaches integrating environmental science, clinical microbiology, and agricultural practices are vital for effectively managing these threats.

Public health strategies should enhance surveillance systems for fungal infections, particularly in vulnerable populations (Casadevall, 2019, 2020). Research efforts should prioritize understanding the environmental determinants of fungal virulence and the development of novel therapeutic and preventive measures.
